# Vitamin D status of adults in Germany

**DOI:** 10.17886/RKI-GBE-2016-042

**Published:** 2016-12-14

**Authors:** Martina Rabenberg, Gert B.M. Mensink

**Affiliations:** Robert Koch Institute, Department for Epidemiology and Health Monitoring, Berlin, Germany

**Keywords:** NUTRITION, VITAMIN D, HEALTH SURVEY, DEGS1, GERMANY

## Abstract

Vitamin D plays an important role in the body as part of bone metabolism. Blood serum concentrations demonstrate that 30.2% of adults (29.7% of women and 30.8% of men) have a deficient vitamin D status. In total, 38.4% of adults (38.6% of women, 38.3% of men) have an adequate status. Although there is little variation among men between the various age groups, the proportion of women with deficient vitamin D status increases with age, while the proportion of women with an adequate status decreases. Furthermore, adults with a low socio-economic status are significantly more likely to have a deficient vitamin D status than adults with a higher socio-economic status. Vitamin D status is subject to strong seasonal fluctuations. In order to ensure adequate concentrations of serum vitamin D, it is recommended to expose the face, hands and arms to the sun two to three times a week between March and October without using sun protection; however, sunburn should be strictly avoided.

## Introduction

Vitamin D is a fat-soluble vitamin that acts in the body like a hormone. An important role in the body is its participation in bone metabolism including promoting absorption of calcium from the small intestine, and strengthening the bones [1]. Severe and prolonged vitamin D deficiency, therefore, can cause bone weakening and skeletal deformations. This may result in rickets in infants and children and osteomalacia in adults.

In older age, vitamin D deficiency can contribute to the development of osteoporosis. In recent years, observational studies have also identified associations between low vitamin D concentrations and various chronic diseases such as type 2 diabetes mellitus, cardiovascular disease and different types of cancer [2–4]. However, evidence of a causal relationship is lacking [5, 6].

The vitamin D status depends on the intake of vitamin D through the diet and the production of vitamin D in the skin when it is exposed to UV-B radiation (vitamin D synthesis) [7, 8]. Since only a small number of foods, such as fatty fish or mushrooms, contain sufficient quantities of vitamin D, the body has to synthesise the vast majority – an estimated share of 80% to 90% – of its vitamin D supply [1]. However, the levels of solar radiation needed to produce enough vitamin D are only available throughout the year at latitudes below 35°. At higher latitudes, the intensity and duration of solar radiation decreases, making vitamin D synthesis dependent on the season in these regions [9–11]. This also applies to Germany, which lies between latitudes 47° and 55°. In Germany, subcutaneous synthesis of vitamin D can take place between March and October [11]. During this time, the body synthesises vitamin D and stores it in fat and muscle tissue and can be used during the winter months. However, various lifestyle factors inhibit the development of an adequate vitamin D reserve (such as staying indoors or a strong sun protection-behaviour). Therefore, low vitamin D levels are relatively common, particularly during the dark winter months.

People who rarely go outside and those who usually cover their skin when they do so (because of reliance on nursing care or for religious or cultural reasons), those who have darker skin, as well as older people have a higher risk of vitamin D deficiency [12]. The same applies to people with chronic gastrointestinal, liver or kidney disease, and to people who take medicines that negatively affect vitamin D metabolism (such as antiepileptic and cytostatic drugs).

## Indicator

The German Health Interview and Examination Survey for Adults (DEGS1) [13, 14], which was conducted by the Robert Koch Institute between 2008 and 2011, assessed the vitamin D status of 6,995 participants aged 18 to 79 years by measuring serum 25-hydroxyvitamin D (25(OH)D) [15].

The Institute of Medicine, USA, has evaluated the possible impact of vitamin D status on bone health [16]. An adequate level of vitamin D was assumed at a 25(OH) D serum concentration of > 50 nmol/l. Serum concentrations between 30 and < 50 nmol/l indicate a suboptimal level of vitamin D and are associated with possible negative consequences for bone health. 25(OH)D serum concentrations of < 30 nmol/l indicate a deficient level of vitamin D which is associated with an increased risk of diseases such as osteomalacia and osteoporosis [11]. In the following, vitamin D status is presented using this classification and according to gender, age, socio-economic status and season.

## Reflection of the results

In total, 30.2% of adults (29.7% of women and 30.8% of men) between the age of 18 and 79 years have 25(OH)D serum concentrations <30 nmol/l and thus a deficient vitamin D status. Only 38.4% of adults (38.6% of women and 38.3% of men) have an adequate status with 25(OH)D serum concentrations ≥50 nmol/l (Tables 1 and 2).

The results stratified for age show that the proportion of women with an adequate vitamin D status significantly decreases with age, whereas the proportion of women with a deficient status increases slightly (Tables 1 and 2). Among men, the age-trend is less clear. Although the percentage of men with a deficient vitamin D status slightly decreases with age, the proportion of men with an adequate status remains almost constant throughout. The gender-specific differences across the age strata are not entirely explainable. Possible causes which have been discussed include women’s higher percentage of body fat and a stronger tendency to seek protection from the sun [17].

The data provided by DEGS1 also demonstrate that women and men with a low socio-economic status significantly more often have a deficient vitamin D status than women and men with a high socio-economic status (Tables 1 and 2). There are also significantly more women in the middle and high groups for socio-economic status with an adequate vitamin D status than women in the lower socio-economic group. Such differences are not observed among men and the reason for this is partly unclear. However, it is likely that differences between people’s behaviour during their leisure time – especially with respect to outdoor activities – significantly contribute to these observations. It is also conceivable that other risk factors associated with vitamin D deficiency are less common among individuals with a high socio-economic status.

**Fig. 1:**
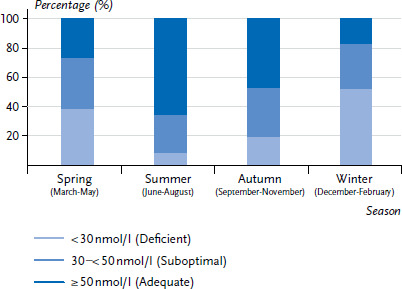
25(OH)D serum concentrations as classified by the Institute of Medicine according to season (n=6,995) Source: DEGS1 (2008–2011)

Figure 1 shows that vitamin D status is subject to strong seasonal fluctuations. During summer, 8.3% of adults have a deficient vitamin D status; during autumn, this is the case for 19.3% of adults. In spring, 38.4% of adults and in winter time, 52.0% of adults have a deficient status. The proportion of adults with an adequate vitamin D status varies similarly strong between the seasons, ranging from 27.3% in spring, to approximately 65.8% in summer, 47.9% in autumn and 17.6% in winter. In addition to the seasons, other factors are known to influence the body’s synthesis of vitamin D. These include the length of time spent in the sun, the use of sunscreen, clothes or other items that cover the body, preferences for shade when outside as well as age and skin pigmentation [11].

The DEGS1 data on adult vitamin D status can be compared with results from the German National Health Interview and Examination Survey 1998 (GNHIES98). GNHIES98 was conducted by the Robert Koch Institute between 1997 and 1999; 25(OH)D serum concentrations of 4,030 participants aged between 18 and 79 years were analysed. A comparison of data from GNHIES98 and DEGS1 shows that the women and men who took part in GNHIES98 had somewhat higher serum 25(OH)D concentrations than those who participated in DEGS1: 23.6% of women and 23.7% of men had a deficient vitamin D status in the GNHIES98 study, with 43.2% of women and 42.7% of men showing an adequate status.

Comparisons of serum 25(OH)D concentrations at the national and international level are influenced and complicated by several factors [11]. These include the different methods used by laboratories to determine 25(OH)D, which can lead to different results [11, 17–20]. In order to make studies on vitamin D status in Europe comparable, 25(OH)D serum concentrations were taken from 14 representative studies (n=55,844), including DEGS1, and calibrated against a reference method using a standardised protocol [11, 21–23]. This was done within the framework of a project funded by the EU (ODIN – Food-based solutions for optimal vitamin D nutrition and health through the life cycle). After standardisation, 44.0% of the participants of DEGS1 (44.3% of women and 43.7% of men) were found to have an adequate vitamin D status, whereas 15.2% (14.7% of women and 15.7% of men) had a deficient vitamin D status [11, 24]. An overall pooled estimate, which was based on all the participating studies, resulted in a vitamin D deficiency prevalence of 11.7% [11]. Consequently, the average serum 25(OH)D concentrations for the population in Germany are lower than the average calculated for all of the participating countries [11]. The highest average serum 25(OH)D concentrations were observed in Finland, presumably due to the increased fortification of foods with vitamin D in this country.

In summary, our analyses demonstrate a suboptimal vitamin D status among adults in Germany. In order to counter vitamin D deficiency, especially in the darker months, current recommendations suggest exposing the face, hands and arms to the sun two to three times a week between March and October for some time without the use of sunscreen [25]. In order to enable an adequate level of vitamin D synthesis, the skin needs to be exposed to the sun for about half of the amount of time that would normally lead to sunburn. Since skin redness and sunburn should be strictly avoided, protection is required if the skin is exposed to the sun for longer periods [25].

The German Nutrition Society recommends vitamin D supplementation for adults, only in certain circumstances: in cases of evident vitamin D deficiency; and in cases where an improved vitamin D status cannot be achieved via endogenous synthesis or diet [26]. This is particularly the case with the risk groups for vitamin D deficiency as mentioned in the introduction [12].

## Key statements

- 30.2% of adults have a deficient vitamin D status and 38.4% of adults have an adequate vitamin D status.
- The proportion of women with a deficient vitamin D status increases with age; the proportion of women with an adequate status decreases with age.
- There are only small age-related differences among men.
- Adults with a low socio-economic status are significantly more likely to have a deficient vitamin D status.
- Vitamin D status is subject to strong seasonal fluctuations.

## Figures and Tables

**Table 1:** 25(OH)D serum concentrations for 18- to 79-year-old women as classified by the Institute of Medicine according to age and socio-economic status (n=3,635) Source: DEGS1 (2008–2011)

	25(OH)D < 30 nmol/l (deficient)		25(OH)D 30 - < 50 nmol/l (suboptimal)		25(OH)D ≥ 50 nmol/l (adequate)	
	Women	Total	Women	Total	Women	Total
	% (95% Cl)	% (95% Cl)	% (95% Cl)	% (95% Cl)	% (95% Cl)	% (95% Cl)
**Age**						
18 – 29 years	25.1 (20.7 – 30.0)	28.4 (24.4 – 32.8)	28.4 (24.0 – 33.3)	28.8 (25.4 – 32.4)	46.5 (40.8 – 52.2)	42.8 (38.2 – 47.7)
30 – 44 years	31.8 (26.5 – 37.6)	32.8 (28.2 – 37.8)	25.9 (22.3 – 29.7)	27.2 (24.2 – 30.4)	42.4 (36.5 – 48.4)	40.0 (35.0 – 45.2)
45 – 64 years	28.8 (25.1 – 32.7)	29.6 (26.1 – 33.3)	34.5 (31.5 – 37.6)	32.8 (30.4 – 35.2)	36.8 (32.9 – 40.8)	37.7 (33.9 – 41.6)
65 – 79 years	32.9 (28.3 – 37.9)	30.0 (26.2 – 34.2)	36.9 (32.8 – 41.3)	36.5 (33.2 – 39.9)	30.1 (25.5 – 35.2)	33.5 (29.2 – 38.0)
**Socio-economic status**						
Low	37.6 (31.6 – 43.9)	38.6 (33.5 – 43.9)	37.1 (32.4 – 42.1)	33.5 (29.7 – 37.4)	25.3 (21.0 – 30.2)	28.0 (23.6 – 32.9)
Medium	28.7 (25.3 – 32.4)	29.0 (25.4 – 32.7)	30.5 (28.1 – 33.1)	30.8 (28.6 – 33.1)	40.7 (36.5 – 45.2)	40.2 (36.1 – 44.5)
High	22.8 (18.6 – 27.6)	24.8 (21.0 – 29.2)	30.6 (26.8 – 34.7)	31.3 (28.5 – 34.2)	46.6 (41.3 – 51.9)	43.8 (39.3 – 48.5)
**Total**	**29.7 (26.5 – 33.1)**	**30.2 (26.9 – 33.8)**	**31.8 (29.7 – 33.9)**	**31.3 (29.4 – 33.3)**	**38.6 (35.0 – 42.3)**	**38.4 (34.7 – 42.3)**

CI = confidence interval

**Table 2:** 25(OH)D serum concentrations as classified by the Institute of Medicine for 18- to 79-year-old men according to age and socio-economic status (n=3,360) Source: DEGS1 (2008–2011)

	25(OH)D < 30 nmol/l (deficient)		25(OH)D 30 – < 50 nmol/l (suboptimal)		25(OH)D ≥ 50 nmol/l (adequate)	
	Men	Total	Men	Total	Men	Total
	% (95% Cl)	% (95% Cl)	% (95% Cl)	% (95% Cl)	% (95% Cl)	% (95% Cl)
**Age**						
18 – 29 years	31.6 (26.1 – 37.6)	28.4 (24.4 – 32.8)	29.1 (24.6 – 34.1)	28.8 (25.4 – 32.4)	39.3 (33.3 – 45.7)	42.8 (38.2 – 47.7)
30 – 44 years	33.8 (28.0 – 40.2)	32.8 (28.2 – 37.8)	28.5 (24.0 – 33.4)	27.2 (24.2 – 30.4)	37.7 (31.6 – 44.2)	40.0 (35.0 – 45.2)
45 – 64 years	30.4 (25.9 – 35.3)	29.6 (26.1 – 33.3)	31.1 (27.8 – 34.6)	32.8 (30.4 – 35.2)	38.5 (33.6 – 43.6)	37.7 (33.9 – 41.6)
65 – 79 years	26.6 (21.8 – 32.2)	30.0 (26.2 – 34.2)	36.0 (31.5 – 40.7)	36.5 (33.2 – 39.9)	37.4 (32.0 – 43.1)	33.5 (29.2 – 38.0)
**Socio-economic status**						
Low	39.6 (33.0 – 46.7)	38.6 (33.5 – 43.9)	29.6 (24.1 – 35.7)	33.5 (29.7 – 37.4)	30.8 (24.3 – 38.2)	28.0 (23.6 – 32.9)
Medium	29.2 (24.8 – 34.0)	29.0 (25.4 – 32.7)	31.1 (28.2 – 34.2)	30.8 (28.6 – 33.1)	39.7 (34.9 – 44.7)	40.2 (36.1 – 44.5)
High	26.5 (21.6 – 32.0)	24.8 (21.0 – 29.2)	31.9 (28.0 – 36.0)	31.3 (28.5 – 34.2)	41.6 (36.0 – 47.6)	43.8 (39.3 – 48.5)
**Total**	**30.8 (26.8 – 35.2)**	**30.2 (26.9 – 33.8)**	**30.9 (28.4 – 33.6)**	**31.3 (29.4 – 33.3)**	**38.3 (33.8 – 42.9)**	**38.4 (34.7 – 42.3)**

CI = confidence interval
